# Misdiagnosis of renal pelvic unicentric Castleman disease: a case report

**DOI:** 10.3389/fsurg.2023.1225890

**Published:** 2023-08-31

**Authors:** Dian Fu, Bo Yang, Ming Yang, Zhenyu Xu, Wen Cheng, Zhijia Liu, Liming Zhang, Zhiguo Mao, Cheng Xue

**Affiliations:** ^1^ Department of Urology, Jinling Clinical Medical College, Nanjing Medical University, Nanjing, China; ^2^ Division of Nephrology, Changzheng Hospital, Second Military Medical University, Shanghai, China; ^3^ Internal Medicine III (Nephrology), Naval Medical Center of PLA, Second Military Medical University (Naval Medical University), Shanghai, China; ^4^ Department of Nephrology, Shanghai Fourth People’s Hospital, School of Medicine, Tongji University, Shanghai, China; ^5^ Department of Urology, 8th medical Center of the PLA General Hospital, Beijing, China; ^6^ Department of Nephrology, Zhabei Central Hospital of JingAn District of Shanghai, Shanghai, China

**Keywords:** Castleman disease, renal tumor, diagnosis, computerized tomography, imaging

## Abstract

Castleman disease is a rare heterogeneous lymphoproliferative disorder of unknown etiology. Unicentric Castleman disease (UCD) is more common. UCD can occur at any site where lymphatic tissue exists, most commonly in the mediastinum, neck, and abdominal cavity, etc. in the current study, we reported a 46-year-old woman, who has left low back pain and discomfort. Magnetic resonance imaging (MRI) of the kidneys showed the left renal pelvis was occupied, left hydronephrosis, and the left renal hilum and retroperitoneal lymph nodes were enlarged. Enhanced kidney CT showed that the “pelvic tumor” was moderately enhanced in the bottom part in corticomedullary phase, while in nephrogenic phase, it was unevenly enhanced with a highly enhanced bottom part and weakly enhanced upper part. In excretory phase, reinforcement was decreased. “left renal pelvis tumor” was diagnosed and she underwent surgical treatment with left nephrectomy. However, histopathological examination indicated the UCD. We suggest that for renal pelvic tumors having imaging characteristics of homogeneous soft tissue density and heterogeneous CT enhancement, the hyaline vascular type of UCD could be taken into consideration for differential diagnosis.

## Background

Castleman disease also known as angiofollicular lymph node hyperplasia, is a rare heterogeneous lymphoproliferative disorder of unknown etiology (1). First described by Dr. Benjamin Castleman in the 1950 s, this disorder is characterized by the abnormal overgrowth (hyperplasia) of lymphoid tissue, leading to the development of lymph node masses. While the majority of cases are localized and non-life-threatening, there are also systemic forms of the disease that can be more severe and challenging to manage. Three basic pathologic types of this disease could be encountered: hyaline vascular type, plasma cell type, and mixed type. Castleman disease also has been classified into various subtypes based on clinical characteristics, including unicentric and multicentric forms.

Unicentric Castleman disease (UCD) is more common (about 90%), hyaline vascular histopathologic is the leading subtype, and in most cases has no obvious symptoms. The symptoms of the disease are often related to the direct compression of the surrounding tissue (2). Surgical excision can improve the prognosis of patients (3). UCD can occur at any site where lymphatic tissue exists, most commonly in the mediastinum, neck, abdominal cavity, etc. Pararenal localizations are very rare and have been reported to account for 2% of cases (4). In the current study, we reported a 46-year-old woman who had a rare UCD in the renal pelvis, which was easily misdiagnosed as renal pelvis tumors. When renal pelvic tumors had imaging characteristics of homogeneous soft tissue density and heterogeneous CT enhancement, the hyaline vascular type of UCD could be taken into consideration for differential diagnosis.

## Case report

**Figure 1:**
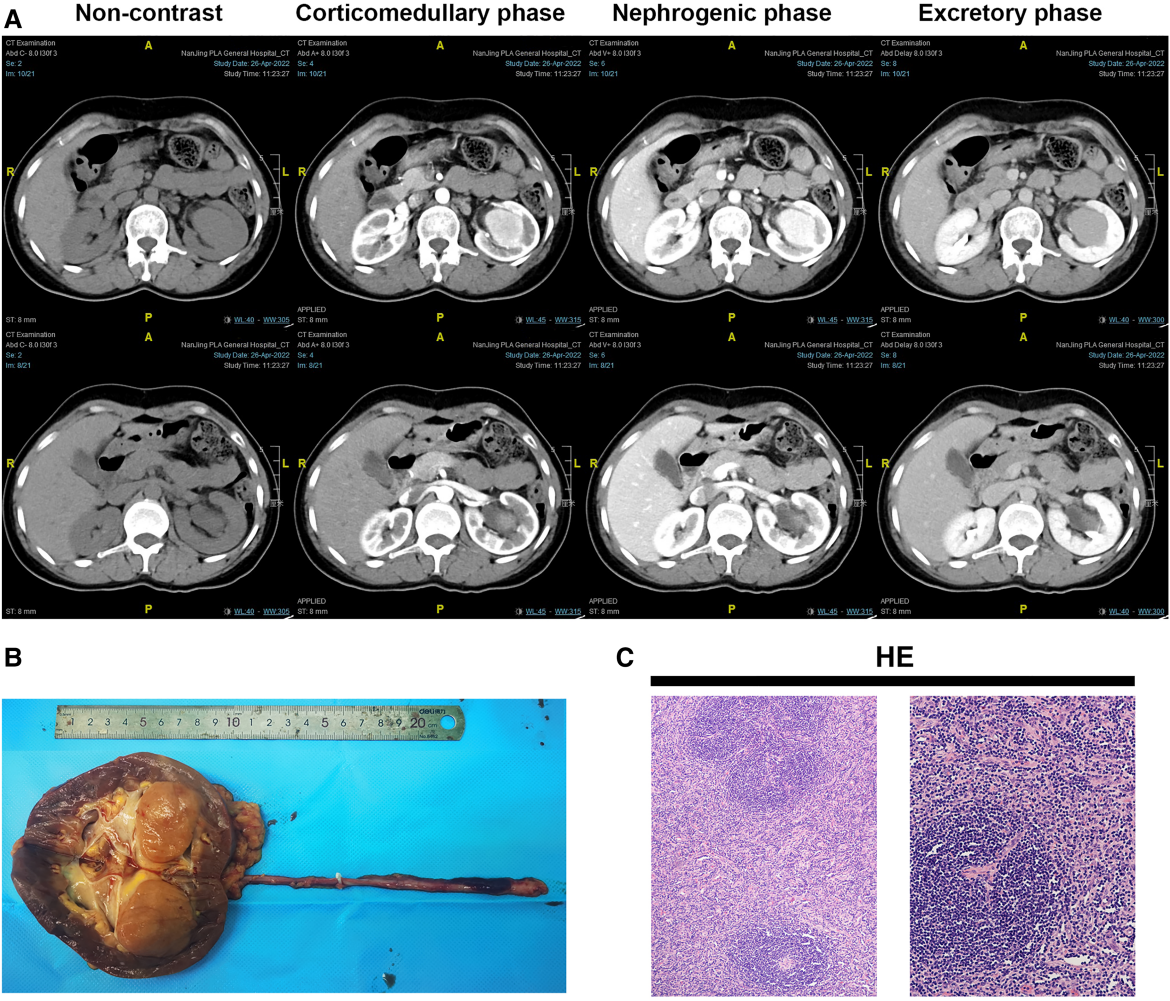
Gross view of the left kidney, HE panel, and abdominal enhanced CT scan of the patient. (**A**) Gross view of the left kidney and ureter; (**B**) HE panel of the lesion. HE staining showed diffusely proliferating lymphoid tissue, scattered lymphoid follicles with partial atrophy, increased blood vessels between follicles, swelling of endothelial cells, and thickening of the vessel wall with hyaline degeneration. The mantle zone has multiple layers of small lymphocytes arranged in a ring, forming a centripetal ring structure, showing an “onion skin”-like structure (the left picture, X100). The blood vessels between the follicles vertically insert into the germinal center to form a “lollipop"-like follicle (the right picture, X200). (**C**) The first column was the lower part of the kidney in enhanced CT.

A 46-year-old woman with no known past medical history presented to the urology department with left low back pain and discomfort for 2 weeks on 6 May 2022. CRP was 0.4 mg/L. Levels of ESR, PCT, and IL-6 were all within the normal ranges. Human herpes virus 8 (HHV-8), HIV, HBV, HCV, and syphilis tests were all negative. Urinalysis test and hematuria routines were normal. The patient underwent abdominal computed tomography (CT) scan and found that the left renal pelvis was spherically enlarged and filled with a soft tissue density shadow. Further examination is recommended. The patient then underwent magnetic resonance imaging (MRI) of both kidneys. The results showed the left renal pelvis was occupied, the likely diagnosis was a pelvic tumor, left hydronephrosis, and the left renal hilum and retroperitoneal lymph nodes were enlarged. For differential diagnosis, the patient underwent enhanced kidney CT. The “pelvic tumor” was moderately enhanced in the bottom part in the corticomedullary phase, while in the nephrogenic phase, it was unevenly enhanced with a highly enhanced bottom part and weakly enhanced upper part. In the excretory phase, reinforcement was decreased (Figure 1A). The patient was not accompanied by fever, abdominal pain, gross hematuria, frequent urination, urgent dysuria, etc. The patient was diagnosed with “left renal pelvis tumor” and underwent surgical treatment with a left nephrectomy. Laparoscopic nephroureterectomy was performed by retroperitoneal approach.

However, in histopathological examination of the left kidney including “tumor” + ureter resection specimens, Castleman disease of the kidney (hyaline vascular type), also known as giant lymph node hyperplasia, was diagnosed with a lesion (size 3.7 cm × 3.5 cm × 3.5 cm, Figure 1B). The lesion involved the renal parenchyma. However, there was no involvement of Castleman disease tissue in the kidney pelvis, perirenal fat capsule, and ureteral resection margin. HE staining showed diffusely proliferating lymphoid tissue, scattered lymphoid follicles with partial atrophy, increased blood vessels between follicles, swelling of endothelial cells, and thickening of the vessel wall with hyaline degeneration (Figure 1C). The immunohistochemistry (IHC) panel showed CD20 (+), CD79a (+), CD3 (+), CD43 (+), CD5 (+), CD21 (+), CD23 (+), CD10 (+), Bcl-2 (3+), Bcl-6 (-), CyclinD1 (-), Ki-67 expression about 10% (+). In situ hybridization of Epstein-Barr virus-encoded RNA (EBER) was negative. Polymerase Chain Reaction (PCR) detection of B lineage gene rearrangement revealed negative IGH and IGK monoclonal bands. The CT of the whole body was performed on the chest and abdomen, and there were no abnormally enlarged lymph nodes and no abnormalities in other parts. PET-CT was not performed after the operation, because it wasn't malignant. Finally, the patient was diagnosed with UCD after systemic Castleman disease was ruled out by clinical systemic examination. Following the nephrectomy, the patient's clinical symptoms improved.

There was no significant change in body weight at one year's follow-up. Tumor biomarkers, such as AFP, CEA, cytoplasmic thymidine kinase 1, and squamous cell carcinoma antigen were all within the normal ranges. Follow-up was performed at 1 month, 3 months, 6 months, 9 months, and 12 months after operation, respectively. All serum and urinary indicators were within normal ranges. Preoperative serum creatinine (scr) was 58.3 µmol/L, while postoperative scr was 84.9 µmol/L, 79.4 µmol/L, and 82.3 µmol/L at 1 month, 9 months, and 12 months, respectively.

## Discussion

The presented case of a 46-year-old woman with left low back pain and discomfort highlights the diagnostic challenges and unexpected findings in the evaluation of renal abnormalities. Initially, the patient's symptoms and imaging findings raised suspicion of a pelvic tumor with left hydronephrosis. Like most patients with renal pelvis tumors, there was vague abdominal pain resulting from the abdominal mass. Meanwhile, the mass that compressed the renal pelvis or proximal ureter caused hydronephrosis in this patient. However, further investigations, including MRI and enhanced kidney CT, revealed a different and rare diagnosis: UCD of the kidney, specifically the hyaline vascular type. While Castleman disease typically affects lymph nodes in the chest and abdomen, it can uncommonly involve other sites, including the kidney, as seen in this case.

In this case, the presence of a spherically enlarged left renal pelvis filled with soft tissue density shadow on CT prompted further investigation. The findings from MRI and enhanced kidney CT, such as moderate enhancement in the corticomedullary phase and decreased enhancement in the excretory phase, were consistent with a solid renal mass. Such radiological characteristics could overlap with those of renal cell carcinoma, making the diagnosis more challenging. Diagnostic imaging methods such as ultrasound, CT, and MRI generally are hard to differentiate UCD from other tumors because of lacking specific signs (5). UCD appears as soft tissue density nodules or swelling on plain CT scans with uniform density. In the current case, the lesions were characterized by heterogeneous CT enhancement. The reason for this may be the difference in blood supply from blood vessels in the upper and lower part of the lesion. The characteristic pathology of the hyaline vascular subtype is the proliferation of a large number of small blood vessels in and between the follicles, so a rich capillary network is formed inside the lesion. Even though renal biopsy can be undertaken to assess the tumor malignancy and to help to avoid extensive resection, most surgeons rarely perform the biopsy of an enhancing renal mass (6). Therefore, we suggest that for renal pelvic tumors having characteristics of homogeneous soft tissue density and heterogeneous CT enhancement, the hyaline vascular type of UCD should be taken into consideration.

Histopathological examination and immunohistochemistry played a crucial role in reaching the correct diagnosis. The characteristic features of Castleman disease, including the presence of germinal centers and hyalinized vessels, were observed in the lesion. The immunohistochemistry panel showed the expression of various markers, such as CD20, CD79a, CD3, CD43, CD5, CD21, CD23, CD10, and Bcl-2, which helped distinguish Castleman disease from other conditions like lymphomas. Importantly, HHV-8 and *in situ* hybridization for EBER was negative, ruling out an association with the virus, which can sometimes be implicated in multicentric Castleman disease. Additionally, PCR detection of B lineage gene rearrangement revealed negative results for IGH and IGK monoclonal bands, further supporting the diagnosis of UCD (7).

The differentiation between unicentric and multicentric Castleman disease is crucial, as the latter carries a more systemic involvement and may require different treatment approaches. In this case, the patient's clinical symptoms significantly improved following the left nephrectomy, which is a standard treatment for localized UCD (7).

The rarity of Castleman disease and its atypical presentation in the kidney underscore the importance of considering this condition in the differential diagnosis of renal masses. This case report adds to the existing literature on the variable manifestations of Castleman disease and emphasizes the value of imaging and histopathological analysis in arriving at an accurate diagnosis. Since it is rare, few previously published studies reported cases of CD involving the renal sinus (Table 1) (8–15). The presentation of the patients is unspecific in all of the reported cases. The leading subtype was UCD, and the leading histopathological subtypes were HV/PC. What makes this disease interesting is that it has only been reported in Asian populations. The exact reason is unknown.

**Table 1:** Characteristics of previously reported cases of CD with renal sinus involvement.

Study	Race	Age	Sex	Clinical type	Pathologic type
Li 2019 (11)	Asian	56	M	UCD	Hyaline vascular CD (right kidney)
Zhang 2022 (8)	Asian	40	F	UCD	Hyaline vascular CD (left kidney)
Jang 2012 (9)	Asian	64	M	UCD	Plasma-cell CD (left kidney)
Guo 2019 (10)	Asian	62	F	UCD	Plasma-cell CD (right kidney)
Nagahama 2000 (12)	Asian	79	M	MCD	Plasma-cell CD (left kidney)
Nishie 2003 (13)	Asian	65/70/73	M (*n* = 2) F (*n* = 1)	UCD (*n* = 1) MCD (*n* = 2)	Plasma-cell CD (*n* = 2) mixed form CD (*n* = 1)
Park 2007 (14)	Asian	50	M	MCD	Hyaline vascular CD (right kidney)
Kim 2012 (15)	Asian	59	M	UCD	Plasma-cell CD (left kidney)

M, male; F, female; CD, Castleman disease; UCD, unicentric CD; MCD, multicentric CD.

Overall, this case report serves as a valuable reminder for clinicians to remain vigilant and consider rare entities like Castleman disease when evaluating patients with renal masses, particularly in instances where the typical presentations do not align with the final diagnosis. Further research and case studies are warranted to deepen our understanding of this rare disease and refine the best management strategies for affected individuals.

## Data Availability

The original contributions presented in the study are included in the article/Supplementary Material, further inquiries can be directed to the corresponding author.
